# A Novel Generation of Polysulfone/Crown Ether-Functionalized Reduced Graphene Oxide Membranes with Potential Applications in Hemodialysis

**DOI:** 10.3390/polym14010148

**Published:** 2021-12-31

**Authors:** Andreea Madalina Pandele, Madalina Oprea, Andreea Aura Dutu, Florin Miculescu, Stefan Ioan Voicu

**Affiliations:** 1 Advanced Polymer Materials Group, University Politehnica of Bucharest, 1-7 Gh. Polizu Street, 011061 Bucharest, Romania; pandele.m.a@gmail.com (A.M.P.); madalinna_09@yahoo.com (M.O.); 2 Department of Analytical Chemistry and Environmental Engineering, Faculty of Applied Chemistry and Materials Science, University Politehnica of Bucharest, 1-7 Gh. Polizu Street, 011061 Bucharest, Romania; 3 Faculty of Medical Engineering, University Politehnica of Bucharest, 1-7 Gh. Polizu Street, 011061 Bucharest, Romania; aura.dutu26@yahoo.com; 4 Faculty of Materials Science and Engineering, University Politehnica of Bucharest, 313 Spl. Independentei, 060042 Bucharest, Romania; florin.miculescu@upb.ro

**Keywords:** hemodialysis, covalent functionalization, composite membranes, polysulfone

## Abstract

Heavy metal poisoning is a rare health condition caused by the accumulation of toxic metal ions in the soft tissues of the human body that can be life threatening if left untreated. In the case of severe intoxications, hemodialysis is the most effective method for a rapid clearance of the metal ions from the bloodstream, therefore, the development of hemodialysis membranes with superior metal ions retention ability is of great research interest. In the present study, synthetic polysulfone membranes were modified with reduced graphene oxide functionalized with crown ether, an organic compound with high metal ions complexation capacity. The physico-chemical characteristics of the composite membranes were determined by FT-IR, Raman, XPS and SEM analysis while their efficiency in retaining metal ions was evaluated via ICP-MS analysis. The obtained results showed that the thermal stability of reduced graphene oxide was improved after functionalization with crown ether and that the presence of the carbonaceous filler influenced the membranes morphology in terms of pore dimensions and membrane thickness. Moreover, the ability of Cu^2+^ ions retention from synthetic feed solution was up to three times higher in the case of the composite membranes compared to the neat ones.

## 1. Introduction

Heavy metals are defined as naturally occurring elements, with a relatively high density compared to water, comprising essential (e.g., Cu, Fe, Ni, Zn) and non-essential metals (e.g., Cd, Hg, Pb, As). They are considered trace elements because they are present in very low concentrations (less than 10 ppm) in the environment and also in living organisms [1,2]. Throughout time, heavy metals found applications in industry, agriculture, medicine and technology, this severely altering their geochemical cycles and biochemical balance. These human-related activities, combined with their bio-accumulative potential, lead to raising concerns regarding their potential adverse effects on human health [3]. It was found that the main routes for heavy metal poisoning are industrial exposure, air or water pollution, contaminated medicines, improperly coated food containers, or the ingestion of lead-based paints. Moreover, studies showed that some of the complications of heavy metals toxic effects are gastrointestinal and kidney dysfunction, nervous system disorders, skin lesions, vascular damage, immune system dysfunction, birth defects, and even cancer [4,5]. There are various methods for the removal of heavy metals from the human body, however, if the renal system is severely affected, hemodialysis is the most recommended one [1].

Hemodialysis is an extracorporeal filtration process in which uremic toxins are removed from the blood using a semipermeable membrane [6]. The efficiency of the dialysis process is mainly based on the characteristics and performances of the dialysis membranes. A key feature of these membranes is selectivity-they allow only small water soluble compounds (e.g., urea, creatinine), middle molecules (e.g., β-2 microglobulin, leptin, complement protein), and protein-bond molecules to pass through, while blocking proteins such as albumin, and other larger molecules [7]. Depending on the type of transported molecule, solute transport in the membranes is governed by two mechanisms–diffusion and convection. For example, small water soluble molecules diffuse through the membrane while the transport of protein-bond molecules is driven by convection [8].

Cellulose in the form of collodion, cellophane or cuprammonium rayon was initially used for the production of dialysis membranes [9]. However, studies showed that, upon contact with blood, the hydroxyl groups present on the polymer backbone activate the complement system and initiate coagulation, thus leading to cardiovascular events [10]. For a better performance of cellulosic membranes, thickness reduction, substitution of hydroxyl groups and pore size increase were proposed [11].

Nowadays, synthetic membranes are most frequently used for hemodialysis due to their easily controllable properties [12]. More than that, synthetic membranes usually have larger pores, thus allowing higher ultrafiltration rates and an effective removal of uremic toxins with higher molecular weight [13]. Based on their potential to activate the complement system, polysulfone, acrylonitrile and polyamide were classified as highly biocompatible in comparison with cuprophane (least biocompatible) and cellulose-acetate with medium biocompatibility [14]. Amongst synthetic polymers used for the production of such membranes, polysulfone (PSF) remarks itself in virtue of its appropriate physico-chemical and biological properties such as good solubility in a large range of polar aprotic solvents, high thermal and mechanical resistance, chemical resistance on the entire range of pH and in oxidative medium, intrinsic biocompatibility, high permeability for low molecular weight proteins and high endotoxin retention ability [8]. Polysulfone-based membranes with increased hydrophilicity and ability to suppress hemodialysis-induced oxidative stress were developed by blending PSF with other polymers such as polyethylene glycol [15] chitosan [16] or amino-silanized poly(methyl methacrylate) [17] or by the incorporation of active compounds such as resveratrol [18], silibinin [19] or alpha lipoic acid [20] in the membranes structure.

Polysulfone membranes have certain limitations when they come in contact with blood due to the fouling phenomenon that causes a decline in terms of flow and selectivity. Membrane fouling during hemodialysis is a result of apparent protein adsorption, platelet adhesion, and the activation of clotting enzymes on the membranes surface [21]. The modification of polysulfone membranes can reduce or prevent the fouling phenomenon and can be achieved by blending PSF with other polymers [22,23], functionalizing the polymers surface with various functional groups [24,25] or mixing PSF with different organic or inorganic fillers [26,27], thus resulting mixed matrix composite membranes. These composites synergistically combine the properties of the polymer matrix with the ones of the filler, generating a material with superior properties [28]. Compared to neat polymeric membranes, composite ones present clearer pore channels, higher porosity and show better results in terms of filtration rates and toxin retention [29].

Membrane materials have proven their usefulness and necessity due to their selectivity and large number of practical employments such as water purification by retention of heavy metal ions [30,31,32] and pollutants from the pharmaceutical industry [33,34,35], or biomedical applications [36,37,38,39]. According to various studies, the incorporation of carbonaceous compounds into polymeric membranes increases their thermal stability, improves bioactivity and mechanical properties [4]. In virtue of their high versatility, availability and affordable costs, graphene and graphene derivatives are currently the most employed fillers for the production of composite materials [40]. Graphene is a bi-dimensional sp^2^-hybridized carbon sheet, composed of single carbon atoms, stable in normal conditions [41]. This material presents a growing research interest for a large area of applications due to its superior mechanical, thermal and electrical properties, compared to traditional carbonaceous materials [42]. Graphene’s unique features include high carrier mobility, good optical transparency, high specific surface area, full flexibility and intrinsic biocompatibility, these characteristics making it appropriate for use in the biomedical field [41,43]. Still, the main disadvantage of graphene is that it does not present functional groups on its surface to allow interactions with various polymers, this being the reason why graphene oxide, a graphene derivative was developed [44]. Graphene oxide (GO) is a highly oxidized form of graphene which contains carboxylic groups on the edges and hydroxyl and epoxide groups on the basal plane. These oxygen-containing groups increase the interplanar distance and provide a hydrophilic character to the carbon layers, thus favoring dispersion in water, organic solvents or different polymeric matrices [45,46]. Graphene oxide also presents attractive adsorbent properties due to the interactions that can occur between the negatively charged carboxylic groups and conjugated C−C bond in GO’s structure and the positively charged metal ions in the surrounding environment [47,48]. Another essential feature of graphene oxide is that it can be reduced by thermal or chemical treatments, thus resulting reduced graphene oxide (rGO) with increased electrical conductivity due to its structure similar with pristine graphene [49].

The purpose of this study was the synthesis of a novel generation of composite membranes based on polysulfone and reduced graphene oxide. The membranes were obtained by blending polysulfone with reduced graphene oxide nanoparticles that were previously functionalized with 4′-aminobenzo-15-crown-5 ether (CE) in order to provide them an improved metal ions retention ability. Crown ethers are cyclic molecules that play a crucial role in the formation of host–guest complexes due to their ability to accommodate positive metal ions, coordinated to the ring of oxygen atoms inside their central cavity [50]. It was found that crown ethers can be effectively used for the complexation of copper [51], lead [52,53] and lithium [54] cations. The host-guest complexation mechanism and the ability of crown ethers to selectively recognize and bind specific metal cations from complex mixtures [55] encouraged their utilization in sensing, phase transfer catalysis, extraction, chromatography or biomimetic applications such as membrane-forming amphiphiles and receptors, model ion channels and ionophores [56]. 

In virtue of the favorable characteristics of each component described above, we consider that these novel PSF/rGO-CE membranes could be successfully employed in the case of heavy metals poisoning, when a rapid detoxification of the human body is required, for ”one day hemodialysis” procedures. The novelty degree of this study is represented by the functionalization method that ensures the formation of covalent bonds between the reduced graphene oxide and the complexation agent (CE), thus preventing the release of the active compound in the bloodstream.

## 2. Materials and Methods

Polysulfone with average molecular weight of 35.000 g/mol and pellet form was purchased from Sigma Aldrich (St. Louis, MO, USA) and used as base polymer in the membrane casting solution. N, N-Dimethylformamide (DMF) with analytical purity of 99.8% was purchased from Sigma Aldrich and used as solvent. Tetraethylenepentamine-reduced graphene oxide (rGO-NH2) (Nanoinnova) was employed as functional filler for membrane modification. Cyanuric chloride (CC) and 4′-aminobenzo-15-crown-5 ether (CE), used for the functionalization of rGO-NH2, were obtained from Sigma Aldrich. All substances were used as received without previous purification.

### 2.1. Functionalization of rGO-NH_2_ with CE

**Scheme 1:**
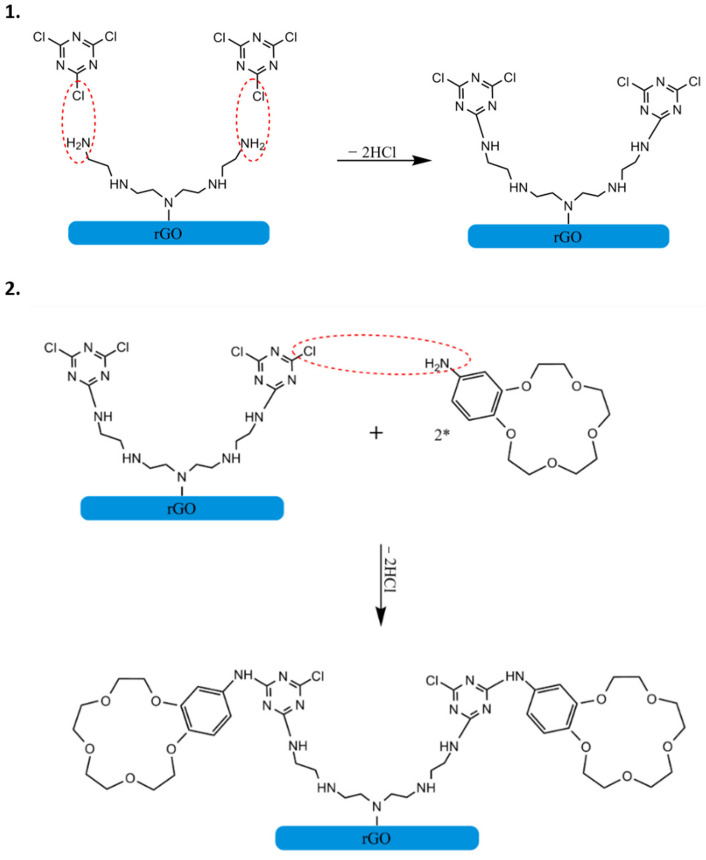
Theoretic reaction mechanism for the functionalization of rGO-NH_2_ with CE.

Tetraethylenepentamine-reduced graphene oxide was chose for this experiment because it contains highly reactive amino (NH_2_) groups in its surface, thus facilitating the functionalization procedure. First, rGO-NH_2_ was dispersed in DMF by ultrasonication at low amplitude for 30 min, on ice bath to prevent overheating. After an even dispersion was achieved, cyanuric chloride was added into the mixture under magnetic stirring, and the temperature was set at 40 °C. Under the influence of the temperature, the chlorine atoms from cyanuric chloride react with the amino groups from rGO-NH_2_ surface forming amine bonds. For the reaction efficacy to be high, the solution was maintained in these conditions for 2 h. Afterwards, the temperature was increased at 70 °C and the crown ether was added in the mixture. The reaction mechanism between the crown ether and cyanuric chloride is similar to the one previously described, more specifically, the amino groups in crown ether’s structure react with the chlorine atoms from cyanuric chloride forming amine bonds. The theoretical mechanism of the functionalization reaction is illustrated in Scheme 1. After 2 h, the dispersion was filtered using a Teflon membrane (0.4 µm pore diameter) and dried in a vacuum laboratory oven for 48 h at 40 °C. The resulting fine, black powder was further subjected to characterization to prove that the functionalization was successful. 

### 2.2. Preparation of the PSF/rGO-NH_2_-CE Composite Membranes

The first step consisted in dissolving the PSF pellets in DMF under magnetic stirring, for 3 h at 50 °C to obtain a 12 wt.% PSF solution. After the complete polymer dissolution, a small amount of functionalized rGO was added (1 wt.%), and the solution was ultrasonicated for 10 min on ice bath to ensure an even filler dispersion. The ultrasonication was realized at low amplitude to prevent the breaking of the bonds formed between the reduced graphene oxide and crown ether. The membranes were then prepared by phase inversion. The phase inversion procedure consisted in casting the cooled polymeric solution on a glass plate and submerging the plate in a coagulation bath containing a non-solvent, in this case distilled water. Due to the solvent and non-solvent exchange, polymer precipitation took place and an asymmetric membrane was formed. The same procedure was followed for the preparation of the neat PSF membrane and both of the resulting membranes were kept in distilled water prior to characterization.

### 2.3. Metal Ions Retention Efficiency

The metallic ions adsorption capacity of the composite membranes was tested using copper sulfate and calcium chloride synthetic feeding solutions with concentration of 1 g/L. A small amount of each feeding solution was kept for analysis, in order to determinate the initial concentration of Cu^2+^ and Ca^2+^. The feeding solutions were then divided in two recipients in which 2 cm × 2 cm samples from the neat and composite membranes were added and kept under magnetic stirring at room temperature. After 4 h, the membranes were extracted and the remaining solutions were analyzed using an Agilent 8800 ICP-MS Triple Quadrupole equipment (Agilent Technologies, Santa Clara, CA, USA). The following equation was used to determine the amount of metal ions retained by the membranes:

(1) $$Q\left[\%\right]=\frac{\left(Ci-Cf\right)}{Ci}\times 100$$

where *Q* is the adsorption capacity expressed in percentages, while *Ci* and *Cf* are the initial and final concentration of metal ions in the tested solutions.

### 2.4. Physico-Chemical Characterization 

Thermogravimetric curves were recorded using a Q500 TA Instruments equipment (TA Instruments, New Castle, DE, USA). The samples were placed in alumina crucibles and heated with 10 °C/min from room temperature (RT) to 800 °C, under nitrogen flow. 

ATR FT-IR spectra were recorded using a Bruker VERTEX 70 spectrometer (Bruker, Billerica, MA, USA), equipped with a diamond ATR device, in the 4000–600 cm^−1^ region, at 4 cm^−1^ resolution. The spectra were recorded as an average of 32 successive measurements for each sample.

Raman spectrometry was conducted using a DXR Raman Microscope (Thermo Fischer Scientific, Waltham, MA, USA) with a laser line of 532 nm, focused by a 10X objective. The spectra were computed based on a number of 10 scans.

Cross-sectional and surface morphology were evaluated using a XL 30 Field Emission ESEM (Philips, Amsterdam, Netherlands) equipped with a high brightness field emission gun operating from 200 V–30 kV with 20 Å resolution digital imaging.

X-Ray microtomography (μCT) was performed on a Bruker SkyScan 1272 microCT (Bruker, Billerica, MA, USA). Rectangular specimens (~2.5 mm length and ~1.5 mm width) were cut from the middle of each membrane. Image acquisition was made with a resolution (pixel size) of 2 μm, a rotation step of 0.4° and 8 average frames per capture. For the reconstruction of the raw images, a Bruker NRecon 1.7.1.6 software package was used and the total porosity and pore size were quantified using the Bruker CTAn analysis software. For that, the 3D reconstructed images obtained from μCT were subjected to a sequence of steps including the selection of a region of interest, image binarization after thresholding and morphometric 3D analysis.

The surface chemistry was studied by X-ray Photoelectron Spectroscopy (XPS) using a K-Alpha instrument from Thermo Scientific (Thermo Fischer Scientific, Waltham, MA, USA), with a monochromated Al Kα source (1486.6 eV), at a bass pressure of 2 × 10^−9^ mbar. Charging effects were compensated by a flood gun and binding energies were calibrated by placing the C 1s peak at 284.4 eV as internal standard. A pass energy of 200 eV and 20 eV were used for survey and high resolution spectra acquisition respectively.

## 3. Results

### 3.1. Characterization of Functionalized rGO-NH_2_

**Figure 1:**
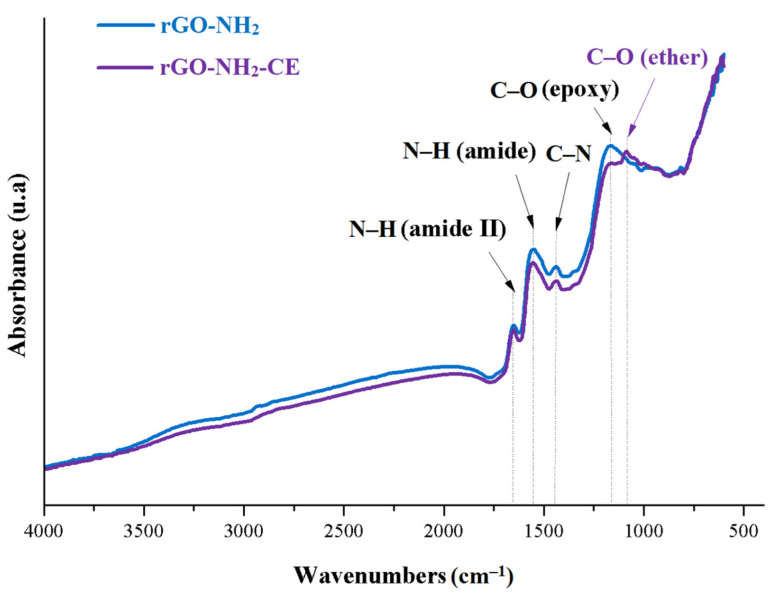
FT-IR spectra of rGO-NH_2_ before and after functionalization with CE.

In order to analyze the chemical changes induced by the functionalization reaction, FT-IR spectroscopy was performed. Figure 1 represents the ATR FT-IR spectra recorded for the neat (rGO-NH_2_) and functionalized reduced graphene oxide (rGO-NH_2_-CE). In both spectra, the peaks characteristic rGO-NH_2_ can be observed. The peaks at 1651 and 1549 cm^−1^ correspond to the N-H bending vibrations from the secondary amide bonded at the reduced graphene oxide surface and the amine groups respectively [22], while the peaks at 1440 cm^−1^ and 1166 cm^−1^ correspond to stretching vibrations of C−N and epoxy groups (C–O–C) in rGO-NH_2_ structure [57]. In the case of rGO-NH_2_-CE, a novel peak is visible at 1087 cm^−1^, value corresponding to the vibrations of C–O bonds from crown ether’s structure, thus confirming that the functionalization reaction was successful [24]. The peaks and their spectral assignments were summarized in Table 1.

**Figure 2:**
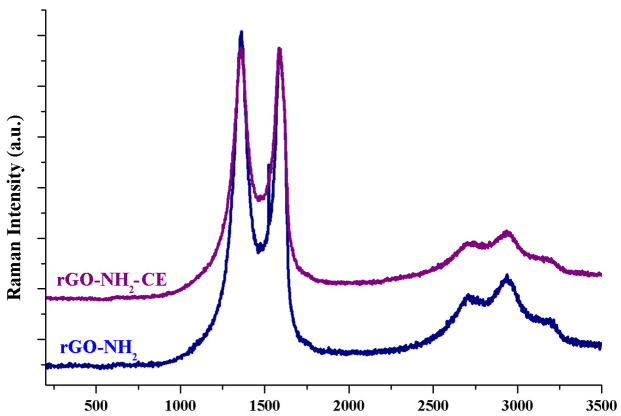
Raman spectra of rGO-NH_2_ before and after functionalization with CE.

Raman spectra were also recorded for the neat and functionalized samples to investigate if the functionalization reaction influenced the disorder degree of reduced graphene oxide. In both spectra (Figure 2), the D and G bands characteristic to graphene-based structures are clearly visible. According to literature, the D band at 1350 cm^−1^ is attributed to the out of plane vibrations attributed to the disordered structure and defects such as vacancies, grain boundaries, and edges in the carbon lattice [58], while the G band at 1580 cm^−1^ corresponds to the in plane vibrations of the sp^2^ bonded carbon atoms [59]. The intensity ratio of D and G peaks (I_D_/I_G_) in the case of functionalized reduced graphene oxide has a similar value to the one obtained for the neat sample, this suggesting that no additional defects were induced in the structure of rGO-NH_2_ during the functionalization reaction [60].

**Figure 3:**
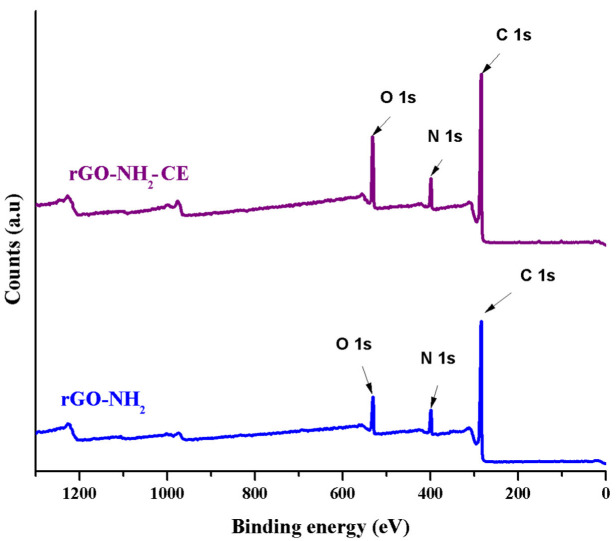
XPS survey spectra of rGO-NH_2_ before and after functionalization with CE.

For a better understanding of the samples surface chemistry, XPS analysis was performed simultaneous, the obtained spectra being provided in Figure 3 and the data are presented in Table 2. In the XPS survey spectra, for both rGO-NH_2_ and rGO-NH_2_-CE, 3 main peaks can be noticed at 285, 399 and 531 eV corresponding to C 1s, N 1s and O 1s respectively. Compared to the neat sample, the functionalized one presents an increased oxygen percentage due to the additional oxygen molecules present in the structure of the bonded crown ether. 

**Figure 4:**
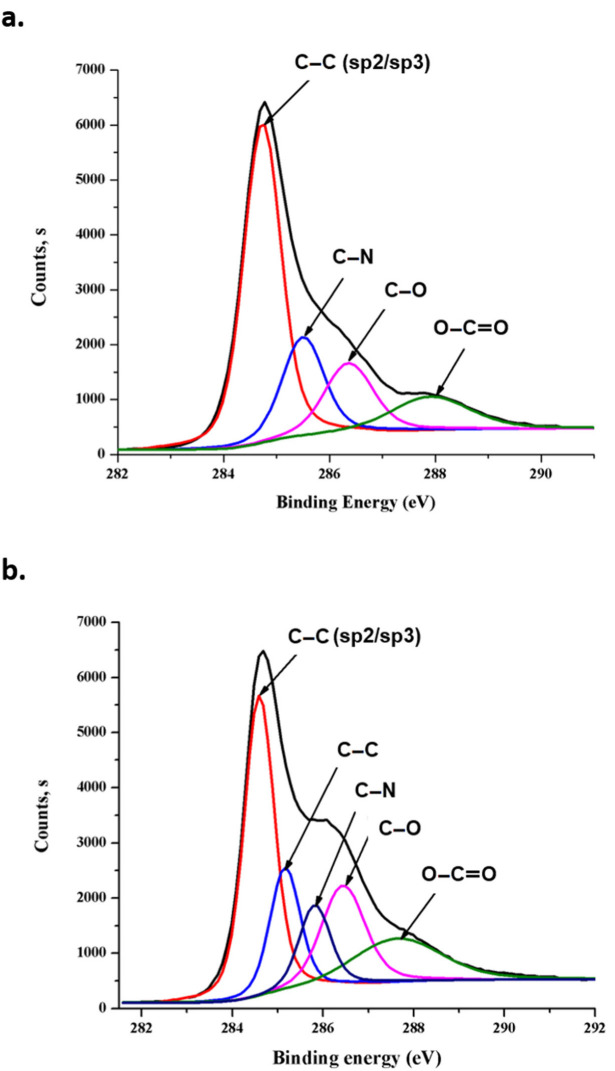
C 1s high resolution XPS spectra of rGO-NH_2_ before (**a**) and after functionalization (**b**) with CE.

The success of the functionalization reaction was also demonstrated by the high resolution C 1s spectrum of rGO-NH_2_-CE (Figure 4) that, besides the bonds characteristic to rGO-NH_2_, also displayed an additional peak at 285.16 eV corresponding to the sp^3^ C–C bonds in CE skeleton [61]. According to previous studies, the other C 1s peaks were attributed to the following types of bonds: 284.73 eV sp^2^/sp^3^ C–C (graphene aromatic structure); 285.5 eV C–N; 286.36 eV C–O (epoxy and alkoxy) and 287.91 eV O–C=O [62,63]. Another interesting fact that can be observed in the C 1s spectra is that the functionalized sample presents an increased number of sp^2^/sp^3^ C–C and C–O bonds correlated with a decrease in the number of C–N bonds, thus suggesting not only that CE is present on the surface of rGO-NH_2_ but also that the functionalization may have occurred at the epoxy groups level [64]. The atom ratio of O/C in rGO-NH2 vs. rGO-NH2-CE is 0.108 vs. 0.168, which also confirm the functionalization with CE.

**Figure 5:**
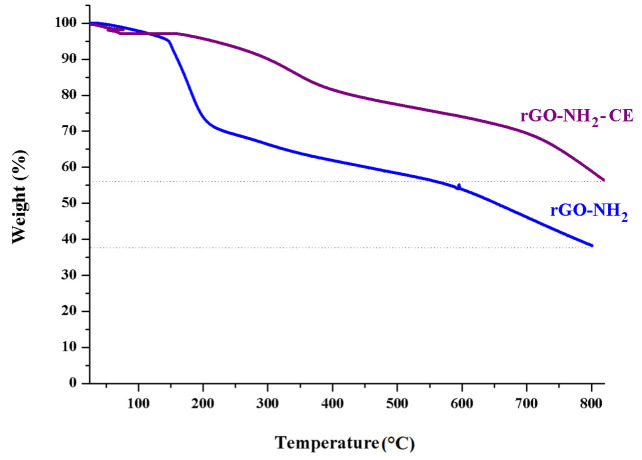
TGA curves of rGO-NH_2_ before and after functionalization with CE.

Thermogravimetric analysis (Figure 5) was realized in order to study the effect of the functionalization on the thermal stability of rGO-NH_2_. The temperature at 10% weight loss (T_10%_), the maximum degradation temperature (T_max_), the residue at 800 °C (R_800_) and the weight loss at 100 °C (WL_100_) were extracted from the thermogravimetric curves and are shown in Table 3. According to the resulting thermogravimetric curves, the neat sample suffered a 32 % weight loss between 100 and 300 °C, with a maximum weight loss at around 179 °C, due to the pyrolysis of the oxygen containing functional groups in reduced graphene oxide’s structure. After reaching the temperature of 600 °C, the observed weight loss was attributed to the degradation of the amino groups present on rGO-NH_2_ surface and to the degradation of the carbonaceous skeleton. The total weight loss recorded at 800 °C was 61.83%. The thermal stability of rGO-NH_2_ was improved after the functionalization with crown ether, both T_10%_ and T_max_ being shifted towards higher values [65]. The rGO-NH2-CE curve illustrates two steps. The first decomposition step, between 24 and 100 °C, is due to the evaporation of residual water molecules, adsorbed on the sample surface. The second degradation step is due to the decomposition of CE molecules attached on rGO’s surface, combined with the combustion of the oxygen containing functional groups [64]. The total weight loss of the functionalized sample was 53.67%, lower compared to the neat one, due to the benzene ring in the crown ether’s structure that protected the carbonaceous skeleton from thermal degradation. Considering these results, the TGA analysis provided another proof that the functionalization of rGO-NH_2_ surface took place.

To summarize the obtained results, it can be said that the additional FT-IR peak at 1087 cm^−1^, corresponding to the C–O bonds in the crown ether structure, and also the increase in the oxygen percentage and the novel C–C peak (285.16 eV) in rGO-NH_2_-CE XPS spectra demonstrated the successful functionalization of reduced graphene oxide. More than that, due to the similarity between the Raman spectra of the neat and functionalized samples, it was concluded that the functionalization reaction did not generate any supplementary defects in the structure of rGO.

### 3.2. Characterization of PSF/rGO-NH_2_-CE Composite Membranes 

**Figure 6:**
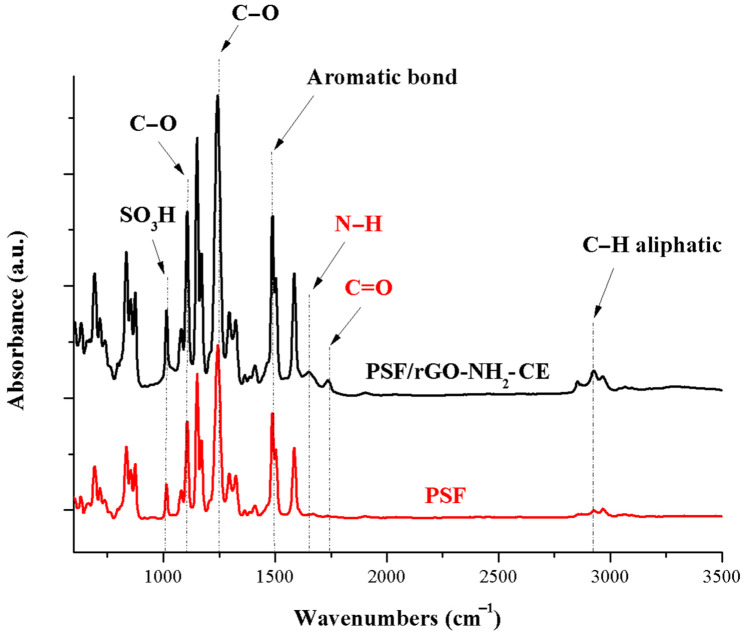
FT-IR spectra of PSF and PSF/rGO-NH_2_-CE.

The peaks observed in the ATR FT-IR spectra (Figure 6) recorded for the neat membrane are in good agreement with the characteristic adsorption bands of PSF reported in literature. More specifically, 1042 cm^−1^ corresponding to S=O stretching vibrations from SO_3_H groups, 1099 cm^−1^ and 1244 cm^−1^ C–O–C stretching, 1149 cm^−1^ O–S–O symmetric stretching in sulfonyl groups (R(SO_2_)-R), 1489 cm^−1^ and 2958 cm^−1^ corresponding to the aromatic ring and aliphatic C–H stretching respectively [66,67]. For the composite PSF/rGO-NH_2_-CE membrane, two additional peaks were observed at 1651 cm^−1^ and 1735 cm^−1^, and were attributed to N–H and carbonyl groups (C=O) in the structure of functionalized reduced graphene oxide [68]. The presence of these groups confirmed the presence of rGO-CE in the structure of the composite membranes.

**Figure 7:**
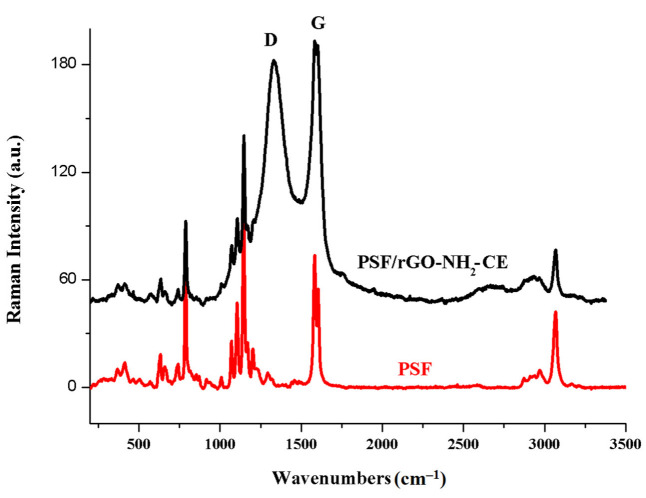
Raman spectra of PSF and PSF/rGO-NH_2_-CE.

The Raman spectra (Figure 7), recorded for both the neat and composite membrane presented peaks at approximately 790 cm^−1^, 1130 cm^−1^, 1600 cm^−1^ and 3063 cm^−1^. According to previous studies, these values are in good agreement with standard PSF [69]. The spectrum obtained for the PSF/rGO-NH_2_-CE composite membrane also contains the D and G bands, characteristic to graphene based structures, at 1350 and 1580 cm^−1^ [67]. The presence of these bands confirms that the carbonaceous filler was successfully included in the polymeric matrix.

**Figure 8:**
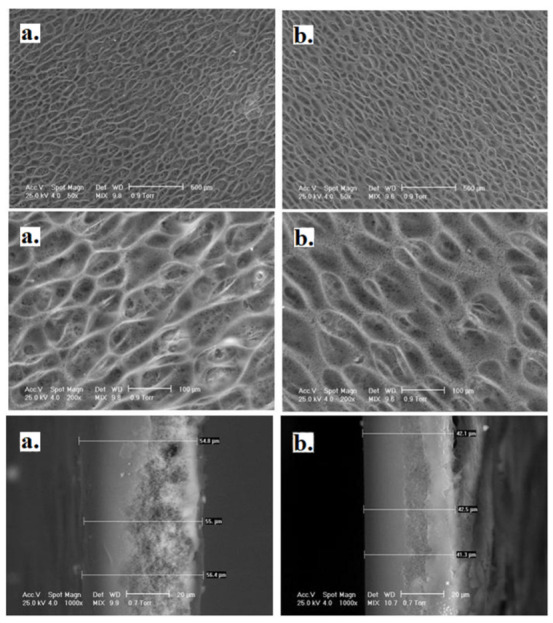
SEM images of PSF (**a)** and PSF/rGO-NH_2_–CE (**b**), top view at two magnification values (50× and 200×), and cross-section (100×).

The SEM images obtained for the porous side of the membranes, at different magnification scales, are shown in Figure 8. At 50× magnification, it can be observed that both PSF and PSF/rGO-NH_2_-CE membranes have a high porosity with interconnected, asymmetric pores. At a higher magnification scale (200×) it is also clear that there are some differences between the neat and composite membrane in terms of pore dimensions, the composite ones having smaller pores. This indicates that the reduced graphene oxide introduced in the composite membranes influenced their morphology and porosity. SEM analysis was also performed in cross-section. The images recorded in transversal section show that the membranes have different thicknesses, even if the same initial quantity of polymer was used. The thickness difference was attributed to the influence of the carbonaceous filler on the polymer’s coagulation process during phase inversion. As the coagulation speed is increased due to the presence of rGO, the solvent is trained to exit more rapidly from the polymeric matrix, thus influencing both the thickness and the porosity of the membrane [70]. Even if its presence was confirmed by previous analysis, the carbonaceous filler was not clearly visible in the SEM images of the composite membranes. This fact was attributed on one hand to the low amount of filler in report to the polymer mass and also to the good dispersion of rGO-NH_2_-CE in the PSF matrix due the ultrasonication treatment.

The porosity and the pore size of the membranes were further calculated using µCT analysis. A decrease of the porosity from 80% in the case of pure PSF membrane to 73% in the case of the composite membrane could be observed. Moreover, as it could also be seen in the SEM images, the pore size of the membranes decreased from 2.5–7 µm to 1.2–4.5 µm after introduction of rGO-NH_2_-CE within the polymer matrix.

**Figure 9:**
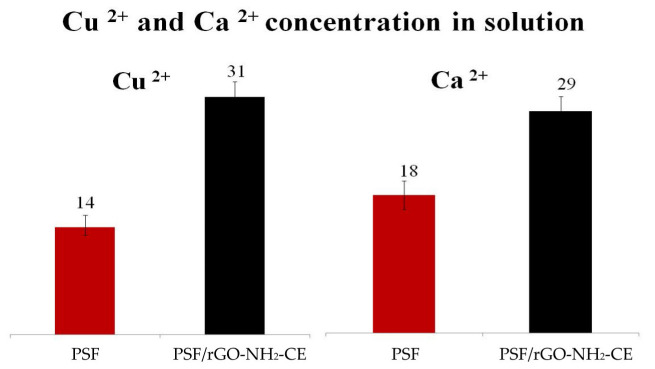
Graphical representation of PSF and PSF/rGO-NH_2_-CE membranes performances in terms of metal ions retention ability.

The metal ions retention ability of the neat and composite membranes was tested using copper sulfate and calcium chloride synthetic feeding solutions (1 g/L concentration). The results obtained following ICP-MS analysis are schematically represented in Figure 9 It can be observed that the neat PSF membrane presented 14% and 18% adsorption efficiency for Cu^2+^ and Ca^2+^ ions respectively, probably due to the electrostatic interactions between the polymer’s surface functional groups and the metal ions and also due to the retention of the metal ions in the membrane pores [71]. However, the composite membranes presented an improved Cu^2+^ and Ca^2+^ ions retention ability, the percentages of adsorbed ions being increased to 31% for Cu^2+^ and 29% for Ca^2+^. Furthermore, the higher retention of copper compared to calcium was attributed to the supplementary interactions between the lone pair of nitrogen from the amine groups of rGO-NH_2_ and Cu^2+^ according to the acid-base Lewis theory [72].

**Figure 10:**
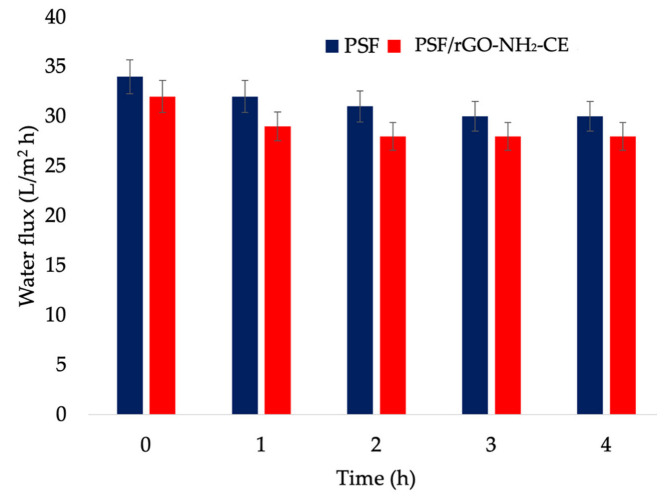
Water fluxes through polysulfone and polysulfone composite membranes after 4 h of recirculation.

Water flux through membranes was evaluated using 500 mL of deionized water under continuous recirculation for 4 h in simulated conditions comparable with medical procedure (Figure 10). After a short decrease (at first hour of recirculation), attributed to the hydrodynamic stabilization of membranes, the composite membrane showed a more stable behaviour, the flux remaining constant in comparison with the neat PSF membrane. This can be explained by the mechanical stabilization of membrane structure given by the presence of the graphene and its high surface area [32]. The measured contact angle was 86.5°, respectively 81.6° for polysulfone, respectively polysulfone composite membrane.

**Figure 11:**
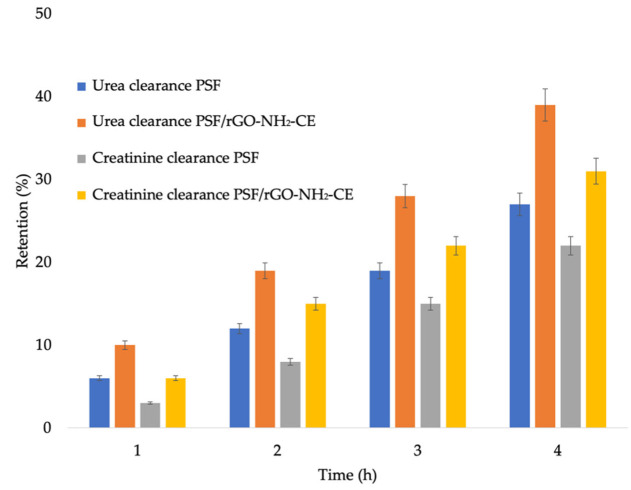
Urea and creatinine clearance through polysulfone and polysulfone composite membranes after 4 h of recirculation.

In order to evaluate the membranes performances for haemodialysis, 250 mL of urea (0.1 mg/mL) and creatinine (0.1 mg/mL) synthetic solutions were used for the clearance of these two uremic toxins evaluation (Figure 11). The rejection for urea increased from 27% for neat polysulfone membrane to 39% for composite polysulfone membrane (an improvement of 44%), while the rejection of creatinine increased from 22% for neat polysulfone membrane to 31% for composite polysulfone membrane (an improvement of 41%), after 4 h of dialysis (in simulated conditions comparable with medical procedure). The obtained results showed a good behaviour of composite membrane with improved capacity in retention of uremic toxins, fact that recommend it for potential use in haemodialysis.

## 4. Conclusions

During this study, composite PSF/rGO-NH_2_-CE membranes were designed for heavy metal retention. Initially, the reduced graphene oxide was functionalized with crown ether, a compound with a high ability to complexate metal ions. The successful functionalization of reduced graphene oxide was demonstrated by FT-IR, Raman, XPS, and TGA analysis. It was observed that the functionalized reduced graphene oxide had a higher thermal stability due to the thermal protection effect of the benzene ring in the structure of the attached crown ether. rGO-NH_2_-CE was further used to prepare composite polysulfone membranes via phase inversion. The presence of reduced graphene oxide in the PSF membrane structure was demonstrated by FT-IR and Raman analysis. The physico-chemical characterization revealed that rGO-NH_2_-CE was identifiable in PSF structure, aspect confirmed by the novel FT-IR peaks at 1651 cm^−1^ and 1735 cm^−1^ attributed to N–H and C=O bonds in the functionalized rGO structure, and also by the presence of D (1350 cm^−1^) and G (1580 cm^−1^) bands in the Raman spectra of the composite membranes. The addition of the carbonaceous filler influenced the thickness and porosity of the PSF membranes, as observed in the SEM images. Metal ion adsorption ability was studied using ICP-MS. The obtained results showed that PSF/rGO-NH_2_-CE composite membranes presented an up to three times higher metal ions adsorption ability compared to the neat PSF ones.

In virtue of their favorable physico-chemical characteristics, it can be said that these novel PSF/ rGO-NH_2_-CE composite membranes could be employed for heavy metal ions retention in “one day hemodialysis” procedures. However, future perspectives consist in testing the PSF/rGO-NH_2_-CE composite membranes in terms of hydrodynamic stability and biocompatibility in order to determine if these membranes indeed present all the requirements for use in biomedical applications. Moreover, the study will be continued by attaching other types of organic molecules on the surface of reduced graphene oxide, depending on the chemical species that are intended to be separated.

## Figures and Tables

**Table 1:** FT-IR peaks spectral assignments.

Chemical Bond	Wavenumber (cm^−1^)
**N–H (amide II)**	1651
**N–H (amine)**	1549
**C–N**	1440
**C–O (epoxy)**	1166
**C–O (ether)**	1087

**Table 2:** XPS atomic percentages of rGO before and after functionalization with CE.

	Atomic Percentage [%]	
	rGO-NH_2_	rGO-NH_2_-CE
**C 1s**	82.97	78.78
**O 1s**	9.02	13.25
**N 1s**	8.01	7.97

**Table 3:** T_10%_, T_max_, R_800_ and WL_100_ values obtained from the thermogravimetric curves.

Sample	T_10%_ [°C]	T_max_ [°C]	R_800_ [%]	WL_100_ [%]
**rGO-NH**_2_	160.26	179.15	38.17	1.95
**rGO-NH**_2_-CE	300.44	344.48	46.33	2.75

## Data Availability

The raw data can be provided at request by the corresponding author of the study.
